# Validation of the predictive value of BDNF -87 methylation for antidepressant treatment success in severely depressed patients—a randomized rater-blinded trial

**DOI:** 10.1186/s13063-024-08061-5

**Published:** 2024-04-09

**Authors:** Hannah Benedictine Maier, Alexandra Neyazi, Gabriel L. Bundies, Fiona Meyer-Bockenkamp, Stefan Bleich, Hansi Pathak, Yvonne Ziert, Barbara Neuhaus, Franz-Josef Müller, Iris Pollmann, Thomas Illig, Stefanie Mücke, Meike Müller, Brinja Kira Möller, Steffen Oeltze-Jafra, Tim Kacprowski, Jan Voges, Fabian Müntefering, Josef Scheiber, Andreas Reif, Mareike Aichholzer, Christine Reif-Leonhard, Maren Schmidt-Kassow, Ulrich Hegerl, Hanna Reich, Stefan Unterecker, Heike Weber, Jürgen Deckert, Nicole Bössel-Debbert, Hans J. Grabe, Michael Lucht, Helge Frieling

**Affiliations:** 1 Department of Psychiatry, Social Psychiatry, and Psychotherapy, Hannover Medical School, Carl-Neuberg-Str. 1, Hannover, 30625 Germany; 2 Department of Psychiatry and Psychotherapy, Otto von Guericke University Magdeburg (OVGU), Magdeburg, Germany; 3 Institute of Biostatistics, Hannover Medical School, Hannover, Germany; 4 Center for Clinial Trials (ZKS), Hannover Medical School, Hannover, Germany; 5 Department of Psychiatry and Psychotherapy, University Hospital Schleswig Holstein, Kiel, Germany; 6 Department of Genome Regulation, Max Planck Institute for Molecular Genetics, Berlin, Germany; 7 Hannover Unified Biobank, Hannover Medical School, Hannover, Germany; 8 Department of Biomarker Analysis and Development, Fraunhofer Institute for Toxicology and Experimental Medicine ITEM, Hannover, Germany; 9 Peter L. Reichertz Institute for Medical Informatics, Hannover Medical School, Hannover, Germany; 10 Division Data Science in Biomedicine, Peter L. Reichertz Institute of Technische Universität Braunschweig and Hannover Medical School, Braunschweig, Germany; 11 Braunschweig Integrated Centre for Systems Biology, Technische Universität Braunschweig, Braunschweig, Germany; 12 Institut Für Informationsverarbeitung, Leibniz University Hannover, Hannover, Germany; 13 BioVariance GmbH, Tirschenreuth, Germany; 14 Department for Psychiatry, Psychosomatics and Psychotherapy, University Hospital Frankfurt - Goethe University, Frankfurt, Germany; 15 Fraunhofer Institute for Translational Medicine and Pharmacology ITMP, Theodor-Stern-Kai 7, Frankfurt Am Main, 60596 Germany; 16 German Foundation for Depression and Suicide Prevention, Leipzig, Germany; 17 Senckenberg Distinguished Professorship, Department of Psychiatry, Psychosomatics, and Psychotherapy, Goethe Universität Frankfurt Am Main, Frankfurt, Germany; 18 Department of Psychiatry, Psychosomatics and Psychotherapy, University Hospital Würzburg (UKW), Würzburg, Germany; 19 Department of Psychiatry and Psychotherapy, University Medicine Greifswald, Greifswald, Germany

**Keywords:** BDNF, Biomarker, RCT, Major depressive disorder

## Abstract

**Background:**

Brain-derived neurotrophic factor (BDNF) is essential for antidepressant treatment of major depressive disorder (MDD). Our repeated studies suggest that DNA methylation of a specific CpG site in the promoter region of exon IV of the BDNF gene (CpG -87) might be predictive of the efficacy of monoaminergic antidepressants such as selective serotonin reuptake inhibitors (SSRIs), serotonin-norepinephrine reuptake inhibitors (SNRIs), and others. This trial aims to evaluate whether knowing the biomarker is non-inferior to treatment-as-usual (TAU) regarding remission rates while exhibiting significantly fewer adverse events (AE).

**Methods:**

The BDNF trial is a prospective, randomized, rater-blinded diagnostic study conducted at five university hospitals in Germany. The study’s main hypothesis is that {1} knowing the methylation status of CpG -87 is non-inferior to not knowing it with respect to the remission rate while it significantly reduces the AE rate in patients experiencing at least one AE. The baseline assessment will occur upon hospitalization and a follow-up assessment on day 49 (± 3). A telephone follow-up will be conducted on day 70 (± 3). A total of 256 patients will be recruited, and methylation will be evaluated in all participants. They will be randomly assigned to either the marker or the TAU group. In the marker group, the methylation results will be shared with both the patient and their treating physician. In the TAU group, neither the patients nor their treating physicians will receive the marker status. The primary endpoints include the rate of patients achieving remission on day 49 (± 3), defined as a score of ≤ 10 on the Hamilton Depression Rating Scale (HDRS-24), and the occurrence of AE.

**Ethics and dissemination:**

The trial protocol has received approval from the Institutional Review Boards at the five participating universities. This trial holds significance in generating valuable data on a predictive biomarker for antidepressant treatment in patients with MDD. The findings will be shared with study participants, disseminated through professional society meetings, and published in peer-reviewed journals.

**Trial registration:**

German Clinical Trial Register DRKS00032503. Registered on 17 August 2023.

## Introduction

### Background and rationale {6a}

Currently, the treatment guidelines for moderate to severe major depressive disorder (MDD) recommend initiating treatment with an antidepressant drug (AD) [1–3]. However, extensive evidence suggests that only one-third of patients achieve complete symptom remission with the initial AD [4]. Additional treatment options, including switching antidepressants, combining different antidepressants, or adding lithium or antipsychotics, are commonly employed, and some have shown effectiveness [1–3]. Despite these treatment alternatives, approximately 40% of patients fail to respond adequately to AD treatments, resulting in a condition known as (pharmaco-)treatment-resistant depression (TRD). Additionally, 15–25% of patients even suffer from “difficult-to-treat depression” (DTD), which describes a depressive condition that “continues to cause significant burden despite usual treatment efforts” [5].

Treatment options for TRD/DTD encompass stimulation therapies such as electroconvulsive therapy (ECT) and innovative antidepressant agents like esketamine [6, 7]. These therapies have exhibited significant efficacy; however, they are typically administered at later stages of the illness, often following months or even years of inadequate treatment. Consequently, patients endure prolonged or chronic illness, leading to considerable personal and societal burden associated with the disease [8, 9]. Unfortunately, no routine diagnostic marker for stratifying or individualizing treatment decisions in MDD are available today.

Over the past few decades, there has been a significant advancement in the field of precision medicine. Although there is promising potential in the form of numerous biomarkers that require verification or falsification, precision psychiatry has yet to catch up [10, 11]. Precision psychiatry aims to integrate biological and environmental data to customize treatments [11]. In this context, the brain-derived neurotrophic factor (BDNF) plays a crucial role concerning antidepressant treatment for MDD. It is implicated in the pathophysiology of MDD as well as the mechanism of action of antidepressants [12–15]. BDNF exon IV has been extensively studied. It acts in regulating its expression through DNA methylation and histone modifications. It contains promoter elements and controls the production of activity-dependent BDNF [16]. We have consistently demonstrated that the DNA methylation of a specific CpG site within the promoter region of exon IV of the BDNF gene is crucial for the effectiveness of current monoaminergic antidepressant treatments, such as selective serotonin reuptake inhibitors (SSRIs), serotonin-norepinephrine reuptake inhibitors (SNRIs), and others [17–19].

Following an initial study conducted by Tadic et al. [17], in which a potential marker was identified in 44 depressed patients, we were able to replicate and validate this finding in a larger cohort of 199 severely depressed patients from the Early Medication Change study (EMC) [18]. In the EMC study, it was observed that patients exhibiting methylation at the CpG site -87 had an almost threefold higher likelihood of achieving remission than those with an unmethylated CpG site. These results were further supported by in vitro experiments, where the effective signaling of BDNF, stimulated by antidepressant incubation, was observed exclusively in constructs with a methylated BDNF exon IV promoter [17, 19]. Wang et al. [20] reported similar findings in a large sample of Chinese patients. However, there is a lack of prospective data regarding the potential of this marker to impact therapeutic decisions.

If our hypothesis that marker-guided treatment leads to reduced adverse events and medications without compromising treatment efficacy will be confirmed, incorporating this test before prescribing antidepressants could prevent unnecessary medication use and minimize the period of patient exposure to ineffective treatments, potentially saving several weeks or even months. This approach would enhance the likelihood of achieving remission from MDD within a shorter timeframe, thereby significantly alleviating patient suffering and reducing the direct and indirect costs associated with the disease.

Implementing a molecular test result as a determining factor for prescribing an antidepressant would represent a transformative shift in paradigm and pave the way for more precise and personalized treatment approaches for depression.

### Objectives {7}

The objective is to demonstrate that knowing the methylation status (marker group) compared to not knowing it (treatment-as-usual (TAU) group) does not result in a significantly lower remission rate and, at the same time, exhibits a statistically significant decrease in the rate of adverse events (AEs).

### Trial design {8}

The BDNF trial is a prospective, interventional, parallel-group, randomized, rater-blinded diagnostic trial conducted at five university hospitals in Germany. The study’s main hypothesis is to show that knowing the methylation status versus not knowing it does not lead to a significantly lower remission rate while significantly reducing AEs. Patient recruitment takes place within the more extensive multicenter cohort study “P4D—Personalized, Predictive, Precise & Preventive Medicine for Major Depression” (for further details, see: German Clinical Trial Register (DRKS) ID: DRKS00032215).

Within the P4D study, 1000 patients diagnosed with MDD undergo comprehensive assessments and deep phenotyping at two time points: upon hospitalization (baseline; BL (day 0–7)) and on day 49 (± 3) as a follow-up assessment (FU). Out of these 1000 patients, 256 eligible patients will be explicitly enrolled for the BDNF trial.

**Fig. 1:**
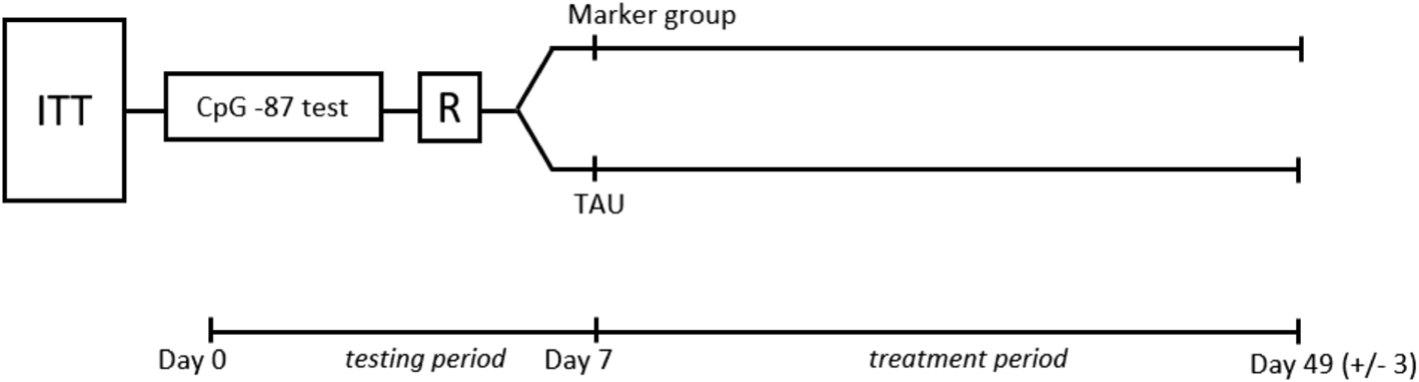
The eligible participants (ITT (intention-to-treat) population) in the BDNF trial, recruited from the five university hospitals, will undergo testing for their BDNF CpG -87 methylation status. Baseline characteristics, including measures such as the Hamilton Depression Rating Scale (HDRS-24) and Montgomery–Åsberg Depression Rating Scale (MADRS) score, will also be collected. Subsequently, participants will be randomly assigned to one of two treatment arms: **a** the Marker group, where test results will be communicated to both participants and treating physicians, and the marker status will be considered in treatment decisions, or **b** TAU (treatment-as-usual) group, where no information about the test result will be provided to participants or physicians, and treatment decisions will be based on the national guidelines [1]

Upon inclusion in the BDNF trial, methylation on BDNF exon IV CpG -87 will be assessed in all patients, and they will be randomly assigned to one of two groups: the *marker group* or the *TAU group*. In the marker group, the test results will be communicated to the patient and their treating physician. Based on this information, treatment decisions will be made collaboratively using a shared decision-making approach. In the TAU group, neither the patients nor their treating physicians will be informed about the methylation status. Treatment decisions for this group will follow the national guidelines (refer to Fig. 1).

All participants of the BDNF trial will receive inpatient treatment for at least 49 days with weekly visits, and a follow-up assessment will be conducted on day 49 (± 3). Additionally, a follow-up by phone will be conducted on day 70 (± 3). Symptomatology as well as the occurrence of AEs will be assessed by raters who are blinded to the participants’ assigned groups. The criterion for the remission of depressive symptoms is indicated by an HDRS-24 sum score equal to or below 10. For evaluating AEs an extended version of the Dosage Record, and Treatment Emergent Side Effects scale (DOTES) is used weekly.

## Methods: participants, interventions, and outcomes

### Study setting {9}

A total of 256 patients will be recruited from five participating university hospitals in Germany: the Department of Psychiatry, Social Psychiatry, and Psychotherapy at Hannover Medical School (MHH; coordinating center), the Department of Psychiatry, Psychosomatics, and Psychotherapy at the University Hospital of Würzburg (UKW), the Department of Psychiatry and Psychotherapy at the University Hospital Schleswig–Holstein (UKSH), the Department of Psychiatry and Psychotherapy at University Medicine Greifswald, and the Department of Psychiatry, Psychosomatic Medicine, and Psychotherapy at the University Hospital Frankfurt (UKF). The study physicians within the P4D cohort will identify and enroll patients. Only patients in the inpatient setting will be eligible for inclusion in the trial.

### Eligibility criteria {10}

Before obtaining informed consent, the inclusion criteria are carefully reviewed and documented (Table 1). Patients who do not meet all the inclusion criteria or meet any exclusion criteria will be excluded from participating in the study. Patients who meet the criteria will be asked to sign a paper consent form, which will be stored at the respective study site.

**Table 1:** Inclusion and exclusion criteria

**Inclusion criteria**	
Age	≥ 18– ≤ 70
Sex	Male, female, or diverse
Major depressive episode according to ICD-10 (F32.2–3, F33.2–3)	Single or recurrent episodes, as confirmed by the DIPS
Symptom severity	Severe (MADRS ≥ 34)
Modification in the therapeutic regimen is being considered	e.g., change of AD or change to neuromodulatory therapy
First MDD episode < 50 years	
Capacity for consent	
Written informed consent	
**Exclusion criteria**	
Acute suicidality	
Accommodation according to state law	(e.g., §16/17 Nds. PsychKG)
Persistent depressive disorder (PDD) (ICD-10: F34.1)	
(Lifetime) diagnoses of	Dementia, schizophrenia, schizoaffective disorder, bipolar disorder, substance addiction with currently necessary detoxification
Simultaneous participation in other clinical trials that are not conducted within the scope of the P4D project	

*Abbreviations: AD* antidepressant drug(s), *AE* adverse event, *ICD-10* 10th revision of the International Statistical Classification of Diseases and Related Health Problems, *MADRS* Montgomery–Åsberg Depression Rating Scale, *MDD* major depressive disorder, *Nds.* PsychKG Lower Saxony Law on Assistance and Protective Measures for the Mentally Ill

### Who will take informed consent? {26a}

The research team will notify eligible patients about the study. Either study physicians or study psychologists will discuss the study’s background and procedure with the patients. Information sheets and consent forms will be given to the patients, and the study physician or psychologist will secure written informed consent from each participant.

### Additional consent provisions for collection and use of participant data and biological specimens {26b}

After obtaining the signed informed consent, ethylenediaminetetraacetic acid (EDTA) whole-blood sample will be collected for DNA purification and testing of the BDNF CpG -87 marker in compliance with international standards and necessary quality assurance systems. After analysis of the marker, the remaining portion of the samples, blood as well as DNA, will be discarded. To date, no additional studies using the generated data and/or biological specimens are planned.

## Interventions

### Explanation for the choice of comparators {6b}

For evaluating the effect of knowing the BDNF -87 methylation status the best way forward is to compare the remission rate and the AE rate in the marker group (treating physicians and patients know the marker status) with a group of patients, who receive TAU (treating physicians and patients not knowing the marker status). In the TAU group, patients and physicians will select subsequent treatments based on national guidelines and the physician’s experience, following the common practices on the ward.

### Intervention description {11a}

For more details, please see the “Trial design {8}” section. The BDNF CpG -87 marker stands out as the initial predictive indicator for non-responsiveness to monoaminergic antidepressants, making it unique with no established gold standard or comparator. Within the study protocol, no specific treatment protocol is provided, allowing the marker to be tested under real-world conditions.

### Criteria for discontinuing or modifying allocated interventions {11b}

Given that there is no study drug intake or intervention involved, aside from the knowledge of the marker status, discontinuation of allocated interventions will not be required during the diagnostic trial.

### Strategies to improve adherence to interventions {11c}

In the current study, study nurses, physicians, and psychologists will monitor inpatients on a weekly basis until the follow-up period. Consequently, patients will benefit from additional attention and care from the study team to ensure adherence to the study protocol.

### Relevant concomitant care permitted or prohibited during the trial {11d}

This intervention occurs within a real-world setting, and as such, no concomitant care or interventions are excluded.

### Provisions for post-trial care {30}

No provisions for post-trial care are provided, as there is no anticipated risk of harm associated with the patient’s participation in the study. Nonetheless, patients are covered by insurance under an accident insurance policy.

#### Compensation

Participating patients are not receiving any form of compensation.

#### Patient and public involvement

Patient participation is a crucial component of the study as it allows the research consortium to address and respond to the needs and concerns of patients regarding the novel biomarker-based treatment approach. By involving patients, prejudices, and misconceptions can be thoroughly discussed and directly addressed during the recruitment process and communication of study findings. In the project’s initial year, the German Depression Foundation (GDF) will conduct a quantitative study to evaluate the beliefs, opinions, knowledge, barriers, and concerns surrounding the use of blood-based biomarkers to guide treatment decisions for depression. This assessment will encompass patients with MDD as well as the general population. The obtained results will serve as a basis for developing informative materials that will be provided to participating patients, as well as for guiding ongoing training for all participating medical doctors, focusing on essential considerations when offering the use of blood-based biomarkers. Through collaboration with the German Alliance Against Depression, the GDF will engage a Patient Advisory Board (PAB) that will be involved in all stages of the proposed study. The PAB’s responsibilities will include, among others, contributing to the design of the quantitative study, shaping information materials, discussing interim study findings, and reviewing public releases based on their expertise and lived experience. By participating in the project’s activities, patients will be enabled to engage in important processes related to their individual health and well-being. Active PAB members will also receive a small monetary allowance for their contribution, and their travel expenses will be reimbursed.

### Outcomes {12}

#### Primary outcome

Two co-primary endpoints are used for evaluating the predictive value of BDNF -87 methylation. The 1st co-primary endpoint is the rate of patients achieving remission during therapy, defined as a score of ≤ 10 on the Hamilton Depression Rating Scale (HDRS-24) on day 49 (± 3). The HDRS-24 is commonly used in clinical trials for depression and remission is considered the most relevant outcome for patients. Achieving remission indicates a higher likelihood of full recovery, improved quality of life, and reduced risk of experiencing a chronic-recurrent form of depression. This endpoint focuses on the absence of residual symptoms rather than just a partial response.

The 2nd co-primary endpoint is the rate of AEs. For evaluating AEs an extended version of the DOTES is used on a weekly basis. If a patient has at least one AE with an intensity of three within the time interval of 49 (± 3) days, it is counted for the co-primary endpoint.

### Participant timeline {13}

During BL, patients will undergo a short-structured interview using the MINI-DIPS (Mini Diagnostic Interview for Mental Disorders) to screen for mental disorders. The chapter on MDD from the Diagnostic Interview for Mental Disorders (DIPS) will be used to characterize the symptoms of depression further. Demographic information, such as sex, age, education, and employment, will also be recorded.

Routine blood work, including inflammation markers, lipid markers, and vitamin status, will be performed at baseline and on day 49 (± 3). Medication intake, symptom severity (assessed using MADRS and HDRS-24), and DOTES will be assessed weekly. The BDI-II will be administered biweekly according to the instructions provided (Table 2).

**Table 2:** Overview of examinations during the BDNF trial

	STUDY PERIOD					
TIMEPOINT	Screening (day 0)	Baseline (day 0-7)	Weekly examinations	Biweekly-examinations	Follow Up (day 49 +/- 3)	Follow Up by phone (day 70 +/-3)
ENROLMENT:						
Eligibility screen	X					
Informed consent	X					
Allocation		X				
ASSESSMENTS:						
MINI-DIPS (+ MD-DIPS)		X				
Demographics (sex, age, education, employment, social network, marital status, living situation)		X				
Routine blood work (HDL, LDL, Triglyceride, cholesterol, differential blood count, HbA1c (only BL), CRP, 25-OH-Vitamin D3, Vitamin B12, folate, cortisol, TSH, Insulin, Na, K, Ca, Cl, CK, eGFR, Kreatinin, GOT, GPT, gammaGT)		X			X	
BDNF blood withdrawal		X				
Current medication and side effects		X	X		X	X
Psychotherapy		X	X		X	X
Other treatment modalities		X	X		X	X
BDI-II		X		X	X	X
MADRS		X	X		X	X
HDRS-24		X	X		X	X
DOTES		X	X		X	X

### Sample size {14}

To calculate the required sample size for the study, assumptions regarding the prevalence for biomarker-positive patients, as well as the rates for the co-primary endpoints in both treatment groups, must be established.

#### 1st co-primary endpoint (remission rate)

Based on the studies by Tadic et al. [17] and Lieb et al. [18], the prevalence of biomarker-positive patients and the remission rate (dependent on the biomarker status) were estimated. Due to the different sample sizes of both studies (Tadic et al.: *n* = 39 and Lieb et al.: *n* = 146), an overall rate was calculated using weighting. Tadic et al. (unpublished data) reported a biomarker-positive rate of 0.38, while Lieb et al. reported a rate of 0.58, resulting in a weighted rate of 0.54. Furthermore, Lieb et al. observed a remission rate of 0.45 for biomarker-positive patients, and Tadic et al. observed a rate of 0.4, leading to a weighted rate of 0.41. For biomarker-negative patients, Lieb et al. reported a remission rate of 0.09, and Tadic et al. reported a rate of 0.23 resulting in a weighted rate of 0.19. Based on these empirical data, the following assumptions were made for the planned study:

1. Rate for biomarker-positive patients: 0.5
2. Remission rate for biomarker-positive patients: 0.4
3. Remission rate for biomarker-negative patients: 0.2

To test for non-inferiority of the remission rate, an equal remission rate of 0.30 in both treatment groups is assumed for sample size calculation. This is derived from the 50:50 ratio of biomarker-positive and biomarker-negative patients, averaging the rates described in points 2 and 3. A two-sided 5% significance level and a non-inferiority margin of − 0.2 (favoring the TAU group) are employed for sample size calculation. Based on these assumptions, a sample size of 222 patients, allocated equally (1:1 allocation ratio) between the treatment groups, is required to achieve 90% power to reject a remission rate difference (marker − TAU group) of less than − 0.2. Selecting a non-inferiority margin of 20% ensures sufficient precision, as demonstrating a significant reduction in AEs (the second co-primary endpoint) is crucial for establishing treatment success.

#### 2nd co-primary endpoint (AE rate)

In a meta-analysis conducted by [21], which examined the efficacy of SSRIs and the incidence of AEs based on placebo-controlled studies, the median AE rate was 0.8 in the treatment group. In this trial, it is assumed that knowing the marker status will result in a reduced use of antidepressants, leading to fewer AEs during pharmacotherapy. Conservatively, it is assumed that the AE rate will be half in the biomarker-negative group compared to the rate in patients treated with antidepressants (AE rate in antidepressant-treated patients: 0.8, AE rate in patients not treated with antidepressants: 0.4). Therefore, it is assumed that the AE rate in the marker group is 0.6, calculated as the mean of the expected AE rates stated. Since all MDD patients in the TAU group receive antidepressant treatment, an AE rate of 0.8 is assumed. A sample size of 218 achieves 90% power to detect a rate difference of − 0.20 (marker − TAU group) for the assumed AE rates, with a one-sided type I error of 2.5%.

To demonstrate the value of knowing the marker status for both co-primary endpoints with sufficient robustness, the final sample size was determined based on the 1st primary endpoint, which requires a larger sample size (*n* = 222 vs. *n* = 218). To compensate for potential treatment effect reduction due to study dropouts, the sample size is increased by 15% to ensure a conservative sample determination (*n* = 256). Sample size calculation was performed with nQuery 9.3.

### Recruitment {15}

Patient recruitment occurs within the larger multicenter cohort study “P4D—Personalized, Predictive, Precise & Preventive Medicine for Major Depression” (for additional information, refer to the German Clinical Trial Register (DRKS) ID: DRKS00032215). Briefly, individuals diagnosed with depression will undergo screening upon admission to a psychiatric ward at any of the five participating university hospitals.

## Assignment of interventions: allocation

### Sequence generation {16a}

The marker status information will be transmitted to the respective study sites’ electronic data management system based on the randomization of the study participants. Patients randomized into the TAU group will not have access to their marker status. Randomization occurs after analyzing the marker status using subject data, which helps to guide the stratification process. The randomization list, incorporating sex, methylation status, and center as stratification characteristics, is created by the Institute of Biometry at MHH and implemented by the data management team in the eCRF. The eCRF is facilitated using the electronic data capture system MARVIN (XClinical). To maintain the blinding of raters, the marker status will be made available to the platform members via the electronic data management system once the diagnostic study is completed.

### Concealment mechanism {16b}

Allocation concealment will be guaranteed, as the randomization code will not be generated until the patient has completed all essential baseline measurements within this diagnostic trial.

### Implementation {16c}

The study physician at each site will receive notification once the randomization for a patient is finalized. The allocation will be disclosed to the patient and the treating physician, but it will remain concealed from the rater who is blinded to the study group.

## Assignment of interventions: blinding

### Who will be blinded {17a}

Assessments will be performed by psychiatrists who are not otherwise participating in the study to maintain blindness to the study group. Patients will be advised before each rating session not to disclose whether they belong to the marker group or not.

### Procedure for unblinding if needed {17b}

Given that our trial involves a marker rather than a drug, there will be no need for emergency unblinding.

## Data collection and management

### Plans for assessment and collection of outcomes {18a}

Patient data will be pseudonymized and recorded in an electronic case report form (eCRF). Patients will either receive a tablet (iPad) for self-administration of self-report questionnaires (e.g., Beck’s Depression Inventory II; BDI-II), or the rater will conduct the assessment and enter the corresponding responses directly into the eCRF (e.g., MADRS). Third-party ratings (MADRS, HDRS-24) will also be collected via eCRF. The administration of the scales can be conducted using a tablet or pen and paper, as per the rater’s preference with subsequent data transfer.

### Plans to promote participant retention and complete follow-up {18b}

Patients will be in the psychiatric ward for a minimum of 49 days. Participation in this diagnostic trial will only impact treatment if the marker status is known. However, the decision on the chosen treatment strategy will ultimately rest with the treating physician and the patient. The study protocol does not provide guidance on the treatment.

In the event of withdrawal from the study, a brief retention follow-up, including MADRS, HDRS-24, DOTES, and current treatment modalities, will be conducted. To ensure data quality for the co-primary endpoints, a phone follow-up will occur on day 49 ± 3, collecting MADRS, HDRS-24, DOTES, and information on current treatment modalities.

### Data management {19}

Clinical data is recorded using the electronic data capture system Marvin (XClinical), which complies with European data protection legislation, specifically the General Data Protection Regulation (GDPR; Regulation (EU) 2016/679). The collected data will be transferred to the P4D Cloud hosted by Leibniz Universität Hannover (LUH). Access to this data is restricted solely to members of the P4D consortium. Since the uploaded data is pseudonymized, de-pseudonymization is not feasible, except at the site where the data is collected. Any distribution of data and rights for analysis and publication requires the approval of the steering committee.

### Confidentiality {27}

Personal data will be handled in compliance with the GDPR. Access to the data will be limited to study investigators and authorized personnel, controlled by appropriate authentication methods.

### Plans for collection, laboratory evaluation, and storage of biological specimens for genetic or molecular analysis in this trial/future use {33}

After obtaining the signed informed consent documentation, patients will undergo assessments following the study protocol, and EDTA whole-blood samples will be immediately sent to Fraunhofer ITEM (Hannover, Germany) for methylation analysis using nanopore sequencing (Oxford Nanopore Technologies plc). The assay will be used to measure reliably and reproducibly the DNA methylation at the CpG -87 locus. The measurements can be completed within days providing timely results for clinical decision making. Fraunhofer ITEM will conduct the establishment, standardization, and validation of the assay following international standards and necessary quality assurance systems before the start of the diagnostic study. After analysis of the marker, the remaining portion of the samples, blood as well as DNA, will be discarded.

## Statistical methods

### Statistical methods for primary and secondary outcomes {20a}

#### Primary outcome

Two co-primary endpoints have been established, which can be translated into the following pairs of statistical hypotheses:

- 1st Co-primary endpoint:
  - H0: Δ (remission rate marker group − remission rate TAU group) ≤ − 0.2
  - H1: Δ (remission rate marker group − remission rate TAU group) > − 0.2
- 2nd Co-primary endpoint:
  - H0: Δ (AE rate marker group − AE rate TAU group) ≥ 0
  - H1: Δ (AE rate marker group − AE rate TAU group) < 0

The primary analysis will be conducted on the intention-to-treat (ITT) population, which includes all patients evaluated in the group to which they were randomly assigned. In cases of missing data, imputation will be performed by considering them as “Treatment Failures” for the “Response Rate” endpoint or as having “Had at least one adverse event” for the “Adverse Events” endpoint. The primary effect estimator for both outcomes will be the Mantel–Haenszel estimator for rate differences, explicitly calculating the difference between the marker and the TAU group, while considering methylation status, treatment center, and sex as strata. The *p*-value and confidence intervals will be calculated using the Cochran-Mantel–Haenszel test procedure. If the lower bound of the 95% confidence interval is above − 0.2, it indicates that the non-inferiority of the marker group compared to the TAU group in terms of remission rate can be demonstrated.

For the 2nd co-primary endpoint, the study aims to demonstrate the superiority of the marker group over the TAU group regarding the rate of adverse events. This second co-primary endpoint is met if the upper limit of the 95% confidence interval is less than 0.

To consider the study successful, both criteria must be met. First, non-inferiority for the 1st co-primary endpoint needs to be demonstrated, which means that the marker group should not be significantly worse than the TAU group in terms of remission rate, with the lower bound of the 95% confidence interval above -0.2. Second, superiority for the second co-primary endpoint should be demonstrated, indicating that the marker group has a significantly lower rate of AE compared to the TAU group, with the upper bound of the 95% confidence interval below 0.

#### Secondary outcome

The study’s secondary endpoints are analyzed descriptively, and the evaluations follow the same principles as the primary analyses. These secondary endpoints provide additional insights into various aspects related to the study. The critical secondary endpoints include the following: (a) rate of description of AD: this assesses the frequency at which AD is prescribed and described in the study population; (b) changes in therapy during the study duration: this evaluates any modifications or adjustments made to the treatment regimens of the patients throughout the study; (c) rate of specific psychotherapeutic treatment; (d) rate of other treatment options; (e) rate of and reasons for leaving the study early; (f) expectations and attitudes regarding AD treatment and use of biomarkers in participating patients and physicians: this explores the beliefs, opinions, and attitudes of both patients and physicians towards AD treatment and the use of biomarkers in the context of depression; (g) doctor-patient relationship.

#### Data analysis plan

The analyses of the primary and secondary endpoints are described in the study protocol (see **“**Statistical methods for primary and secondary outcomes {20a}**” **section).

### Interim analyses {21b}

Not applicable. Interim analyses are not planned within the study protocol, given the relatively short study period, and blinding will be maintained throughout the study. Termination of the study is unlikely, as there are no new interventions involving novel pharmacological substances or treatments. Nevertheless, study termination may be considered if recommended by the ethics board.

### Methods for additional analyses (e.g., subgroup analyses) {20b}

This is not applicable to our study, as no subgroup analyses are planned.

### Methods in analysis to handle protocol non-adherence and any statistical methods to handle missing data {20c}

In the event that patients choose to discharge themselves from the hospital before their scheduled follow-up visit, they will be requested to perform third-party assessments using the MADRS, HDRS-24, DOTES scales, and report their medication/treatment status on the day of discharge. Additionally, they will be queried about the feasibility of conducting a follow-up over the phone using the same set of questionnaires. However, if missing values occur in one of the two co-primary endpoints, they are addressed in the following way: Missing values in the 1st primary endpoint “Remission Rate” are defined as “Treatment Failures” and missing values for the endpoint “AE rate” are defined as having “Had at least one adverse event.”

### Plans to give access to the full protocol, participant-level data and statistical code {31c}

The study plan is accessible to the public through the German Clinical Trials Register (DRKS-ID: DRKS00032503). For the dataset and statistical code, interested parties may request access from the corresponding author after consultation with the steering committee.

## Oversight and monitoring

### Composition of the coordinating center and trial steering committee {5d}

The trial steering committee are the same as in P4D (NCT06027177; DRKS00032215):

Located at Hannover Medical School (MHH) in Germany, the coordinating center is part of the Department of Psychiatry, Social Psychiatry, and Psychotherapy. Recruiting university hospitals provide a pool of principle investigators and co-principal investigators that make up the trial steering committee. They will provide suggestions for the early termination, modification, or continuation of the study as needed. They are responsible for planning, carrying out, and monitoring the research protocol. In addition to overseeing data entry, investigators are in charge of recruiting patients and making sure the study is conducted correctly. The data manager is responsible for maintaining data integrity, guaranteeing data quality, and arranging data acquisition. Within the P4D consortium, meetings are held every 2 weeks.

### Composition of the data monitoring committee, its role and reporting structure {21a}

In this study, there will be no Data Monitoring Committee, as it is not subject to pharmaceutical law regulations.

### Adverse event reporting and harms {22}

AEs in the diagnostic trial are gathered through weekly assessments using an extended version of the DOTES scale. This scale specifically addresses the most common AEs associated with drugs, ECT, repetitive transcranial magnetic stimulation (rTMS), and intranasal esketamine. Investigators also have the option to report any other adverse events through free-text input.

### Frequency and plans for auditing trial conduct {23}

The Center for Clinical Trials will conduct a site visit to each recruiting center once during the recruitment phase. Moreover, members of the coordinating center will visit each recruiting center before enrolling the first patient to ensure harmonization.

### Plans for communicating important protocol amendments to relevant parties (e.g., trial participants, ethical committees) {25}

Any alterations to the research protocol are implemented with the consensus of collaborating stakeholders and require approval through an amendment by the leading ethics committee of MHH, followed by approval from all involved ethics committees. Once approved, these changes will be communicated and documented in the online protocol registrations. All investigators will be promptly notified of the modifications.

### Dissemination plans {31a}

The proposed study protocol and its results will be disseminated through various communication channels (such as information material, press releases/briefings, publications, conferences/symposia, meetings of interest groups, and social media) throughout the entire project duration. A local coordination office will be established to ensure effective coordination among all participating facilities, dissemination of newly acquired knowledge, and the promotion of precision medicine in routine practice using the newly developed assays. This office will be supported by the GDF and the PAB. The collaboration between clinical, biological, and data scientists, along with a company experienced in providing clinical decision tools, and the GDF as the leading particular interest group for MDD patients, creates a strong network for the rapid translation and dissemination of the findings from the BDNF trial.

A dissemination and communication plan will be developed at the outset, outlining the relevant stakeholder groups and their specific needs and strategies to engage with them. Various dissemination tools will be explored to generate awareness and visibility for the project and effectively communicate the project’s outcomes. These activities will involve all project partners and target the scientific community, clinicians, medical practitioners, regulatory bodies, patient groups, and the general public. Each target group will be approached in a tailored manner to meet their specific requirements. The dissemination plan will be a dynamic document that will be continuously monitored and updated to ensure its relevance and effectiveness.

### Ethics approval

The trial will be conducted in accordance with the principles of ICH-GCP (International Council for Harmonisation of Technical Requirements for Pharmaceuticals for Human Use—Good Clinical Practice) and the Declaration of Helsinki. Ethical approval for the study has been obtained from the leading ethics committee responsible for MHH (No. 10843_BO_S_2023). Each participating center will submit an ethics application to its respective ethics committee and will not begin recruiting until receiving a positive vote. Patients participating in the study will be required to provide written consent. They will be fully informed about the study protocol, the study intervention (including detailed information about the medical procedure), and the potential access to their medical records by monitors, auditors, and regulatory authorities. The access to medical records will be done in a manner that respects patient confidentiality and complies with relevant regulations. Patients will have adequate time to ask questions and seek clarification before deciding to participate. They will also receive information and provide consent regarding data protection and data confidentiality. Participants have the right to withdraw their consent at any time without any negative consequences for their treatment. The personal data of all study participants will be handled in compliance with the GDPR of the European Union. Data will be pseudonymized before being entered into a GCP-validated electronic data capture system called MARVIN (provided by XClinical).

## Discussion

This innovative and pragmatic RCT aims to evaluate whether knowledge of the biomarker BDNF exon IV CpG -87 is non-inferior to TAU in terms of achieving remission, while also demonstrating a reduced incidence of AEs in severely depressed patients. Our proposed biomarker has already been independently replicated in a Chinese cohort [20], making this investigation crucial for confirming or refuting its diagnostic value.

In addition to the data gathered in this study, several notable strengths exist. Firstly, our study design closely resembles real-life situations by having minimal exclusion criteria (see Table 1). This means that patients included in the study have MDD and might experience comorbidities [22–24]. This is advantageous since RCTs often have strict inclusion criteria, and take place in highly controlled settings. In our study, the treating physician will decide with the individual patient the patient’s treatment course, mirroring real-world scenarios. Furthermore, we are conducting weekly assessments, allowing us to closely monitor the progression of the disease and the improvement of symptoms. If successful, this biomarker can spare patients from prolonged suffering by avoiding the trial-and-error approach to AD treatment. Secondly, our study incorporates an innovative design whereby patients are randomized only after analyzing their marker status. This approach enhances the precision of the study. Thirdly, since this trial is part of a more extensive cohort study (P4D: DRKS-ID: DRKS00032215), the data collected can be utilized for further characterization and stratification based on the biomarkers and other clinical characteristics and physiological features of depressed patients. This promising approach enables further refinement of patient stratification according to their unique clinical characteristics. Lastly, the study involves industrial partners who will handle the commercialization and distribution of the biomarker if successful, facilitating its translation into clinical practice.

In addition to the strengths mentioned above, this study has some limitations that should be acknowledged. The absence of long-term follow-up beyond day 70 (± 3) by phone prevents us from drawing conclusions regarding the extended progression of the disease and the sustainability of response or remission. However, AD response after 10 weeks is rare. Furthermore, it is essential to recognize that patients with the CpG -87 marker may still exhibit non-response to medication, potentially leading to confusion or misunderstanding, as their expectations for a positive response to pharmacological treatment may not align with the need for alternative interventions such as neurostimulation. The study’s design, which grants treating physicians the autonomy to select treatment courses for each patient, introduces variability in treatment protocols, posing a challenge in attributing outcomes solely to the biomarker. Additionally, the study may not sufficiently address or account for variations in patient treatment adherence or compliance. This could impact treatment outcomes and complicate the biomarker’s predictive value interpretation. On the other hand, there is no reason to believe, that adherence could differ between groups.

## Trial status

Ethics approval No. 10843_BO_S_2023; Protocol Version Number: 2.0. Recruitment has not yet started. Recruitment will be completed after approximately 18 months.

## Data Availability

The data will be pseudonymized. For the dataset and statistical code, interested parties may request access from the corresponding author after consultation with the steering committee.

## Abbreviations

AD: Antidepressant drug
AEs: Adverse events
BDI-II: Beck’s Depression Inventory II
BDNF: Brain-derived neurotrophic factor
BL: Baseline
Ca: Calcium
Cl: Chloride
CK: Creatine kinase
CpG: 5′—C—phosphate—G—3′
CRP: C-reactive protein
DIPS: Diagnostic Interview for Mental Disorders
DNA: Deoxyribonucleic acid
DOTES: Dosage Record, and Treatment Emergent Side Effects scale
DRKS: German Clinical Trial Register
DTD: Difficult-to-treat depression
eCRF: Electronic case report form
EDTA: Ethylenediaminetetraacetic acid
eGFR: Estimated glomerular filtration rate
gammaGT: Gamma-glutamyl transferase
GDPR: General Data Protection Regulation
GOT: Glutamate oxaloacetate transaminase
GPT: Glutamate pyruvate transaminase
EMC: Early Medication Change study
FU: Follow-up assessment
GDF: German Depression Foundation
HbA1c: Hemoglobin A1c
HDL: High-density lipoprotein
HDRS-24: Hamilton Depression Rating Scale
ICD-10: 10Th revision of the International Statistical Classification of Diseases and Related Health Problems
ICH-GCP: International Council for Harmonisation of Technical Requirements for Pharmaceuticals for Human Use—Good Clinical Practice
ITT: Intention-to-treat
K: Potassium
LDL: Low-density lipoprotein
LUH: Leibniz Universität Hannover
MADRS: Montgomery–Åsberg Depression Rating Scale
MDD: Major depressive disorder
MHH: Hannover Medical School
MINI-DIPS: Mini Diagnostic Interview for Mental Disorders
Na: Sodium
Nds. PsychKG: Lower Saxony Law on Assistance and Protective Measures for the Mentally Ill
PAB: Patient Advisory Board
P4D: Personalized, Predictive, Precise & Preventive Medicine for Major Depression
rTMS: Repetitive transcranial magnetic stimulation
SNRIs: Serotonin-norepinephrine reuptake inhibitors
SSRIs: Selective serotonin reuptake inhibitors
TAU: Treatment-as-usual
TRD: (Pharmaco)treatment-resistant depression
